# Comprehensive Analysis of Key Genes, Signaling Pathways and miRNAs in Human Knee Osteoarthritis: Based on Bioinformatics

**DOI:** 10.3389/fphar.2021.730587

**Published:** 2021-08-23

**Authors:** Liang Chang, Hao Yao, Zhi Yao, Kevin Ki-Wai Ho, Michael Tim-Yun Ong, Bingyang Dai, Wenxue Tong, Jiankun Xu, Ling Qin

**Affiliations:** ^1^ Musculoskeletal Research Laboratory, Department of Orthopedics and Traumatology, The Chinese University of Hong Kong, Hong Kong, Hong Kong, SAR China; ^2^ Innovative Orthopaedic Biomaterial and Drug Translational Research Laboratory, Li Ka Shing Institute of Health Sciences, The Chinese University of Hong Kong, Hong Kong, Hong Kong, SAR China

**Keywords:** osteoarthiritis, overlapping genes, signaling pathways, miRNAs, bioinformatics

## Abstract

**Background:** Osteoarthritis (OA) is one of the main causes of disability in the elderly population, accompanied by a series of underlying pathologic changes, such as cartilage degradation, synovitis, subchondral bone sclerosis, and meniscus injury. The present study aimed to identify key genes, signaling pathways, and miRNAs in knee OA associated with the entire joint components, and to explain the potential mechanisms using computational analysis.

**Methods:** The differentially expressed genes (DEGs) in cartilage, synovium, subchondral bone, and meniscus were identified using the Gene Expression Omnibus 2R (GEO2R) analysis based on dataset from GSE43923, GSE12021, GSE98918, and GSE51588, respectively and visualized in Volcano Plot. Venn diagram analyses were performed to identify the overlapping DEGs (overlapping DEGs) that expressed in at least two types of tissues mentioned above. Gene Ontology (GO) enrichment analysis, Kyoto Encyclopedia of Genes and Genomes (KEGG) analysis, protein-protein interaction (PPI) analysis, and module analysis were conducted. Furthermore, qRT-PCR was performed to validate above results using our clinical specimens.

**Results:** As a result, a total of 236 overlapping DEGs were identified, of which 160 were upregulated and 76 were downregulated. Through enrichment analysis and constructing the PPI network and miRNA-mRNA network, knee OA-related key genes, such as *HEY1*, *AHR*, *VEGFA*, *MYC*, and *CXCL12* were identified. Clinical validation by qRT-PCR experiments further supported above computational results. In addition, knee OA-related key miRNAs such as miR-101, miR-181a, miR-29, miR-9, and miR-221, and pathways such as Wnt signaling, HIF-1 signaling, PI3K-Akt signaling, and axon guidance pathways were also identified. Among above identified knee OA-related key genes, pathways and miRNAs, genes such as *AHR*, *HEY1*, *MYC*, *GAP43*, and *PTN*, pathways like axon guidance, and miRNAs such as miR-17, miR-21, miR-155, miR-185, and miR-1 are lack of research and worthy for future investigation.

**Conclusion:** The present informatic study for the first time provides insight to the potential therapeutic targets of knee OA by comprehensively analyzing the overlapping genes differentially expressed in multiple joint components and their relevant signaling pathways and interactive miRNAs.

## Introduction

Osteoarthritis (OA) is the most common joint disease, mainly manifesting as pain, limited joint movement, and joint deformity. The risk factors of OA include trauma, aging, obesity, and heredity (Sandell, 2012; Chen et al., 2017a; Chang et al., 2021). During OA development, the entire joint are affected and undergo articular cartilage degeneration, osteophyte formation, subchondral sclerosis, synovitis, and meniscus degeneration, respectively, indicating the complicated and interactive OA pathogenic mechanisms (Chen et al., 2017a). The efficacies of the current treatments for OA in our clinics are limited. In recent years, exploration of disease-modifying osteoarthritis drugs (DMOADs) aiming at alleviating OA symptoms and/or prevent structural progression have drawn much attention. However, the DMOADs under research and development (R&D) and/or clinical trials mainly focus on one of the OA symptoms, such as cartilage degeneration, subchondral bone remodeling, local inflammation, or joint pain, and their potential downstream targets (Latourte et al., 2020). The possibility that newly explored-drug targets have heterogeneous expression profiles in different joint components raises uncertainty of the drug effectiveness. Besides, the R&D of joint component-specific drugs are also limited so far (Latourte et al., 2020).

Based on the rapid development of high-throughput genomics technologies, such as microarray and next-generation sequencing, bioinformatic analysis has been widely used to identify key genes, signaling pathways, and microRNAs (miRNAs) in various musculoskeletal disorders (Kang et al., 2021; Li et al., 2021; Umeno et al., 2021). So far, many studies have identified and explored potential therapeutic targets of OA based on bioinformatic screening. For example, upregulated arginase 2 (ARG2) in OA cartilage was screened out by microarray and further validated to facilitate cartilage destruction *via* upregulating matrix metalloproteinases (MMPs) (Choi et al., 2019). Activated osteochondral turnover, neurogenesis and inflammation in OA bone marrow lesions (BML) were also identified by microarray bioinformatically (Kuttapitiya et al., 2017). Besides, miRNA candidates that have potential as biomarkers and therapeutic targets in OA were identified and validated *via* comprehensively paired miRNA-messenger RNA (mRNA) analysis and functional enrichment analysis (Kung et al., 2018). In addition to identification of potential therapeutic targets, bioinformatics-based bulk sample analysis further helps classify potential OA subtypes for more precise diagnosis and personalized treatments. In recently years, several studies have stepped forward substantially in classifying potential OA subtypes based on bioinformatics (Soul et al., 2018; Yuan et al., 2020). Their surprising discoveries undoubtedly deepen our understandings on knee OA and will facilitate personalized treatments in the future. However, since previous bioinformatic studies mainly focus on one type of joint components as well, investigations on the overlapping DEGs (overlapping DEGs) in different joint components during OA development are still lacking. In 2016, about 5% overlapping DEGs were observed between the DEGs of the synovium and cartilage, while no further analysis was performed on these identified overlapping DEGs (Park and Ji, 2016). Recently, another study observed about 10% overlapping DEGs in OA cartilage and subchondral bone. They identified *IL11* and *CHADL* as two potential therapeutic targets of OA by comparing their identified cartilage-subchondral bone overlapping DEGs with previously identified OA risk genes (Styrkarsdottir et al., 2018; Tuerlings et al., 2021).

Collectively, we believe it is meaningful to comprehensively analyze the key genes that are differentially expressed during OA development in the different joint components, including articular cartilage, subchondral bone, synovium, and meniscus, and their relevant pathways and miRNAs. Such approaches may provide clues to develop adequate treatments for OA by targeting at overlapping differentially expressed genes (DEGs) in different joint components and their relevant miRNAs and signaling pathways. The present study aims at identifying key genes, signaling pathways, and miRNAs in human knee OA by comparing the preexisting gene expression profiles derived from different joint components, including articular cartilage, synovium, subchondral bone, and meniscus. Specifically, the gene expression profiles (GSE) were obtained from the public available Gene Expression Omnibus database (GEO, http://www.ncbi.nlm.nih.gov/geo/). Gene Expression Omnibus 2R (GEO2R) was performed to identify the overlapping DEGs and followed by qRT-PCR validation. Furthermore, functional enrichment analysis, protein-protein interaction (PPI) analysis, and miRNA-mRNA interaction analysis were carried out to identify relevant signaling pathways and interactive miRNAs. This study may shed light on completer and undiscovered pathogenic mechanisms of knee OA development and pave the way toward the identification of new therapeutic targets for further R&D of effective therapies and clinical translation.

## Materials and Methods

### Gene Expression Profiles in Human Knee OA joint Tissues

The gene expression profiling in cartilage, synovial membrane, subchondral bone, and meniscus tissues was obtained from GEO datasets GSE43923 (Klinger et al., 2013), GSE12021 (Huber et al., 2008), GSE51588 (Chou et al., 2013), and GSE98918 (Brophy et al., 2018), respectively. Three degenerated and three intact cartilage samples were retrieved from a human dataset using the Affymetrix Human Genome U133 Plus 2.0 Array platform (GSE43923). Nine normal and ten OA synovial tissue samples were retrieved from a human dataset using the Affymetrix Human Genome U133 Array platform (GSE12021). Twenty OA and five normal medial tibial subchondral bone samples were retrieved from a human study using the Agilent-026652 Whole Human Genome Microarray 4 × 44K v2 platform (GSE51588). Twelve OA and twelve normal meniscus samples were retrieved from a human dataset using the Agilent-072363 SurePrint G3 Human GE v3 8 × 60K Microarray 039494 platform (GSE98918).

### Identifying DEGs

The original gene expression profiles were analyzed by GEO2R (GEO2R, RRID:SCR_016569; https://www.ncbi.nlm.nih.gov/geo/geo2r/?acc=GSE43923, https://www.ncbi.nlm.nih.gov/geo/geo2r/?acc=GSE12021, https://www.ncbi.nlm.nih.gov/geo/geo2r/?acc=GSE51588, https://www.ncbi.nlm.nih.gov/geo/geo2r/?acc=GSE98918) to identify the upregulated and downregulated DEGs in OA joint tissues, respectively. The criteria for a DEG were |log2FC|>1 and adjusted P-value<0.05. The results were visualized in volcano plots.

### Identification of Overlapping DEGs in Human KOA Joint Tissues

Venn diagram (http://bioinformatics.psb.ugent.be/webtools/Venn/;VennDiagram, RRID: SCR_002414) was used to identify upregulated and downregulated overlapping DEGs in the integral joint tissues including cartilage, synovial membrane, subchondral bone, and meniscus. A specific DEG was identified as overlapping DEG when it appeared at least in two of the joint tissues. All the DEGs were identified by comparing gene expression profiles between osteoarthritic and relatively healthy joint tissues.

### Gene Ontology Enrichment and Kyoto Encyclopedia of Genes and Genomes Pathway Analysis

GO enrichment analysis and KEGG pathway analysis were performed on Metascape platform (http://metascape.org/gp/index.html#/main/step1; Metascape, RRID: SCR_016620) (Zhou et al., 2019). Upregulated or/and downregulated overlapping DEGs were listed and followed by “Custom Analysis.” GO enrichment analysis and KEGG pathway analysis were performed with the thresholds of P-value<0.05 and enrichment gene count ≥2.

### Construction of the Protein-Protein Interaction Network

The Search Tool for the Retrieval of Interacting Genes/Proteins (STRING) database (https://string-db.org/; STRING, RRID: SCR_005223) was used to construct the PPI network (Szklarczyk et al., 2021). The overlapping DEGs were mapped to STRING list to perform multiple proteins search and get a PPI network with interaction scores >0.4. Cytoscape V.3.7.2 (Cytoscape, RRID: SCR_003032) was used to visualize the results from the PPI network and perform module analysis. Genes with connectivity degree ≥10 were identified as hub genes (Shannon et al., 2003).

### Module Analysis

Module analysis was performed using the molecular complex detection (MCODE) plugin on Cytoscape platform (MCODE, RRID: SCR_015828; Cytoscape V.3.7.2, RRID: SCR_003032). The parameters set to identify enriched functional modules were as follows: Degree Cutoff = 2, Node Score Cutoff = 0.2, K-Core = 2 and Maxium. Depth = 100. Modules with the MCODE score ≥4 were identified as significant modules and were further evaluated for GO enrichment analysis and KEGG pathway analysis with the thresholds of P-value<0.05 and enrichment gene count >2.

### Construction of the miRNA-mRNA Network

Experimentally validated key gene-related miRNAs were screened out based on key genes identified above by using miRTarBase 8.0 (http://miRTarBase.cuhk.edu.cn/; miRTarBase, RRID: SCR_017355) with strong evidence (Huang et al., 2020). Those miRNAs targeting at least two key genes were identified as key miRNAs and visualized on Cytoscape V.3.7.2 by constructing the miRNA-mRNA network.

### Clinical Specimens Sampling

Clinical specimens of preserved and degenerated cartilage were harvested from osteoarthritis patients undergoing total knee arthroplasty (TKA) surgery. The included patients had no history of chronic diseases, tumors, autoimmune diseases, and viral chronic infections (hepatitis B virus, hepatitis C virus, human immunodeficiency virus). All patients provided informed consent, and this study was approved by the Joint Chinese University of Hong Kong-New Territories East Cluster Clinical Research Ethics Committee (CREC Ref. No: 2013.248). The estimated sample size equaled to three based on a pilot study. A total of three donors (Age: 70.17 ± 3.66; gender: one male and two females; K-L: grade III) were included. The cartilage extracted from hypertrophic and the severely destructed region was classified into degenerated cartilage (DC) group, and the cartilage extracted from the relatively smooth region was classified into preserved cartilage (PC) group. About 0.5–1 g DC and PC samples were collected from each donor respectively. All specimens were stored at −80°C with 1 ml Trizol (Invitrogen, United States) after grinding and homogenizing with liquid nitrogen. TRIzol™ Plus RNA Purification Kit was used for RNA extraction. Briefly, homogenized tissues were followed by phase separation, RNA precipitation, RNA wash, and RNA redissolving according to experimental protocol. During precipitation step, a 1/10 volume of 3M Sodium acetate (Cat No. AM9740, Invitrogen, United States) was added additionally to help RNA precipitation.

### Quantitative Real-Time Polymerase Chain Reaction

The cDNA was synthesized from total RNA by using PrimeScript RT Master Mix (Perfect Real Time) Kit (Takara, Japan). Quantitative real-time PCR (qRT-PCR) was performed in triplicate on a QuantStudio™ 7 Flex Real-Time PCR System (Life Technologies QuantStudio 7 Real Time PCR System, RRID: SCR_020245, United States) by using TB Green Premix Ex Taq II (Tli RNase H Plus) Kit (Takara, Japan). The primers (5′-3′) were ordered from Tech Dragon Ltd. (Hong Kong) and listed in Table 1 The relative expression of each gene was normalized to GAPDH and presented in heatmap after normalization (log10 transformation).

**TABLE 1:** List of primers used in qRT-PCR experiments.

Gene	Sequence (5′-3′)	
	Forward	Reverse
*AHR*	GTA​AGT​CTC​CCT​TCA​TAC​C	AGG​CAC​GAA​TTG​GTT​AGA​G
*CYP1A1*	CAC​AGA​CAG​CCT​GAT​TGA​GCA	GTG​TCA​AAC​CCA​GCT​CCA​AAG​A
*HEY1*	CTG​CAG​ATG​ACC​GTG​GAT​CA	CCA​AAC​TCC​GAT​AGT​CCA​TAG​CAA
*MYC*	GCC​AAG​CTC​GTC​TCA​GAG​AAG	CAG​AAG​GTG​ATC​CAG​ACT​CTG
CXCL12	ACC​GCG​CTC​TGC​CTC​AGC​GAC​GGG​AAG	TGT​TGT​TCT​TCA​GCC​GGG​CTA​CAA​TCT​G
*VEGFA*	CTC​TAC​CTC​CAC​CAT​GCC​AAG​T	GCT​GCG​CTG​ATA​GAC​ATC​CA
*GAPDH*	GGG​GGA​GCC​AAA​AGG​GTC​ATC​ATC​T	GAG​GGG​CCA​TCC​ACA​GTC​TTC

### Statistical Analysis

All data were analyzed using SPSS Statistics 23.0 software (IBM SPSS Statistics, RRID: SCR_019096, Chicago, United States). Two groups (PC and DC) with paired data were assessed by the paired sample t-test. A P-value less than 0.05 (*p* < 0.05) was considered statistically significant and P-values were presented numerically.

## Results

### Identification of DEGs in Human Knee OA Joint Tissues

**FIGURE 1:**
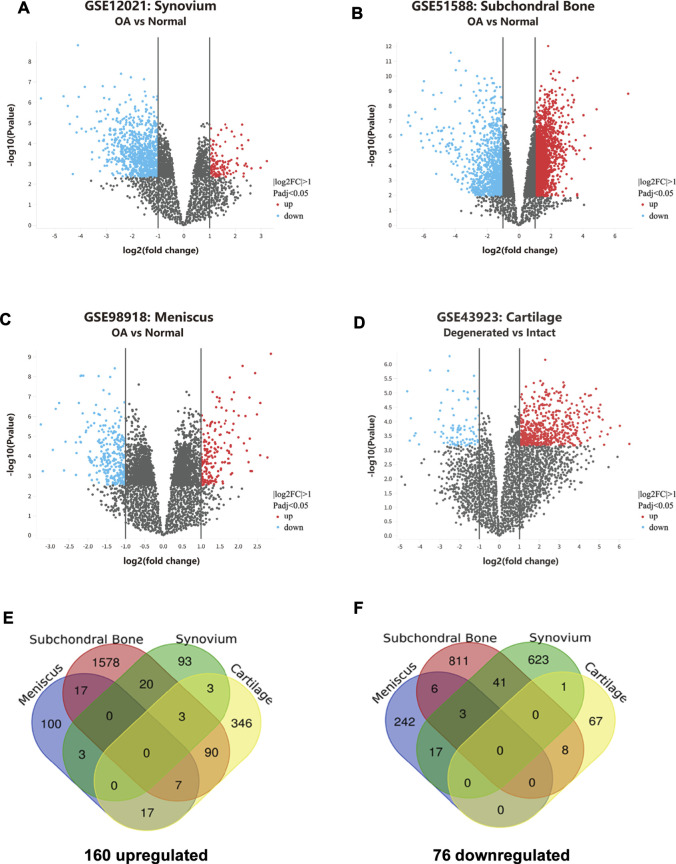
Identification of overlapping DEGs in knee OA. **(A–D)** Gene expression profiles of GSE12021, GSE51588, GSE98918, and GSE43923 are visualized in volcano plots respectively. DEGs are marked with red and the criteria for a DEG are |log2FC|>1 and adjusted P-value<0.05. **(E)** Upregulated DEGs in osteoarthritic cartilage, synovium, subchondral bone, and meniscus. **(F)** Downregulated DEGs in osteoarthritic cartilage, synovium, subchondral bone, and meniscus.

For GSE43923 dataset, a total of 542 genes were identified by GEO2R analysis, of which 466 were upregulated and 76 were downregulated. For GSE12021 dataset, a total of 807 genes were identified by GEO2R analysis, of which 122 were upregulated and 685 were downregulated. For GSE51588, a total of 2,584 genes were identified by GEO2R analysis, of which 1715 were upregulated and 869 were downregulated. For GSE98918, a total of 412 genes were identified by GEO2R analysis, of which 144 were upregulated and 268 were downregulated. The distribution of gene expression for each dataset was visualized in the corresponding volcano plot (Figures 1A–D).

### Identification of Overlapping DEGs in Human Knee OA Joint Tissues

As shown in the Venn Diagrams, 236 overlapping DEGs were identified, of which 160 were upregulated (Figure 1E) and 76 were downregulated (Figure 1F). No overlapping DEG was found in all the OA cartilage, synovial membrane, subchondral bone, and meniscus tissues. Those genes that appeared the most (at least three times) were identified as the most overlapping DEGs and listed in Table 2. A total of 13 most overlapping DEGs were identified. Among them, *AHR*, *HEY1*, *CXCL12*, *MMP9*, *OLFML2A*, *SLITRK6*, *RHBDL2* were highly expressed in cartilage, meniscus, and subchondral bone. *COL8A1*, *GAP43*, and *PTN* were highly expressed in cartilage, synovial membrane, and subchondral bone. In addition, *RUNX1*, *ARL4C*, and *PIM1* were lower expressed in the meniscus, synovial membrane, and subchondral bone.

**TABLE 2:** List of the most overlapping DEGs in OA joint tissues.

Gene	Locations	Expression
AHR	Cartilage, Meniscus, Subchondral Bone	↑
HEY1	Cartilage, Meniscus, Subchondral Bone	↑
CXCL12/SDF1	Cartilage, Meniscus, Subchondral Bone	↑
MMP9	Cartilage, Meniscus, Subchondral Bone	↑
OLFML2A	Cartilage, Meniscus, Subchondral Bone	↑
SLITRK6	Cartilage, Meniscus, Subchondral Bone	↑
RHBDL2	Cartilage, Meniscus, Subchondral Bone	↑
COL8A1	Cartilage, Synovium, Subchondral Bone	↑
GAP43	Cartilage, Synovium, Subchondral Bone	↑
PTN	Cartilage, Synovium, Subchondral Bone	↑
RUNX1	Synovium, Subchondral Bone, Meniscus	↓
PIM1	Synovium, Subchondral Bone, Meniscus	↓
ARL4C	Synovium, Subchondral Bone, Meniscus	↓

AHR, aryl hydrocarbon receptor; HEY1, hairy/enhancer-of-split related with YRPW motif protein 1; CXCL12/SDF1, stromal cell-derived factor 1; MMP9, matrix metallopeptidase 9; OLFML2A, olfactomedin Like 2A; SLITRK6, SLIT and NTRK-like protein 6; RHBDL2, rhomboid Like 2; COL8A1, collagen type VIII alpha 1 chain; GAP43, growth-associated protein 43; PTN, pleiotrophin; RUNX1, RUNX family transcription factor 1; PIM1, proto-oncogene serine/threonine-protein kinase Pim-1; ARL4C, ADP ribosylation factor like GTPase 4C.

### GO Enrichment Analysis

**FIGURE 2:**
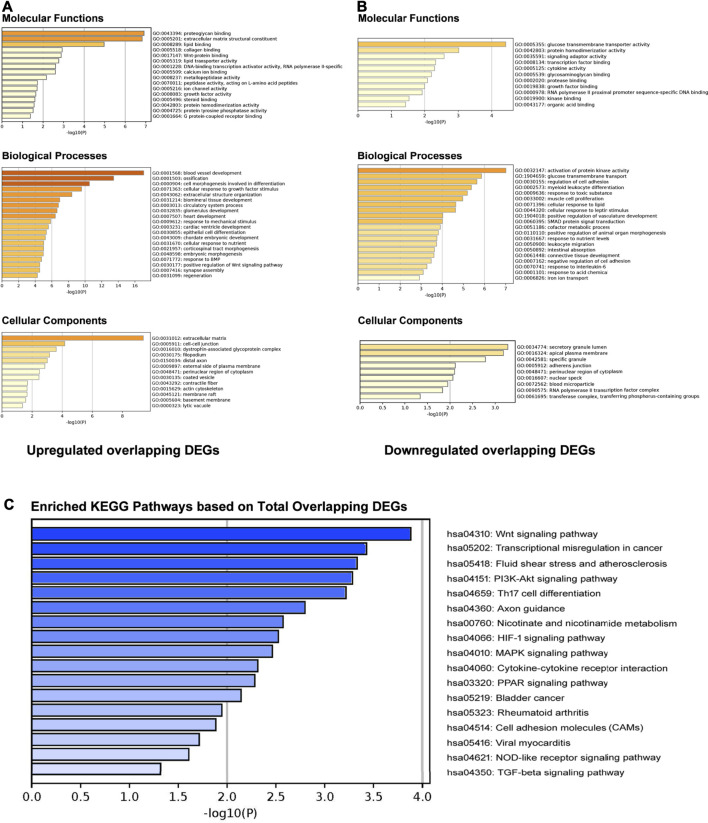
GO enrichment and KEGG pathway analyses. **(A)** GO molecular functions, biological processes, and cellular components enrichment analysis on upregulated overlapping DEGs. **(B)** GO molecular functions, biological processes, and cellular components enrichment analysis on downregulated overlapping DEGs. **(C)** Enriched KEGG pathways based on total overlapping DEGs.

**FIGURE 3:**
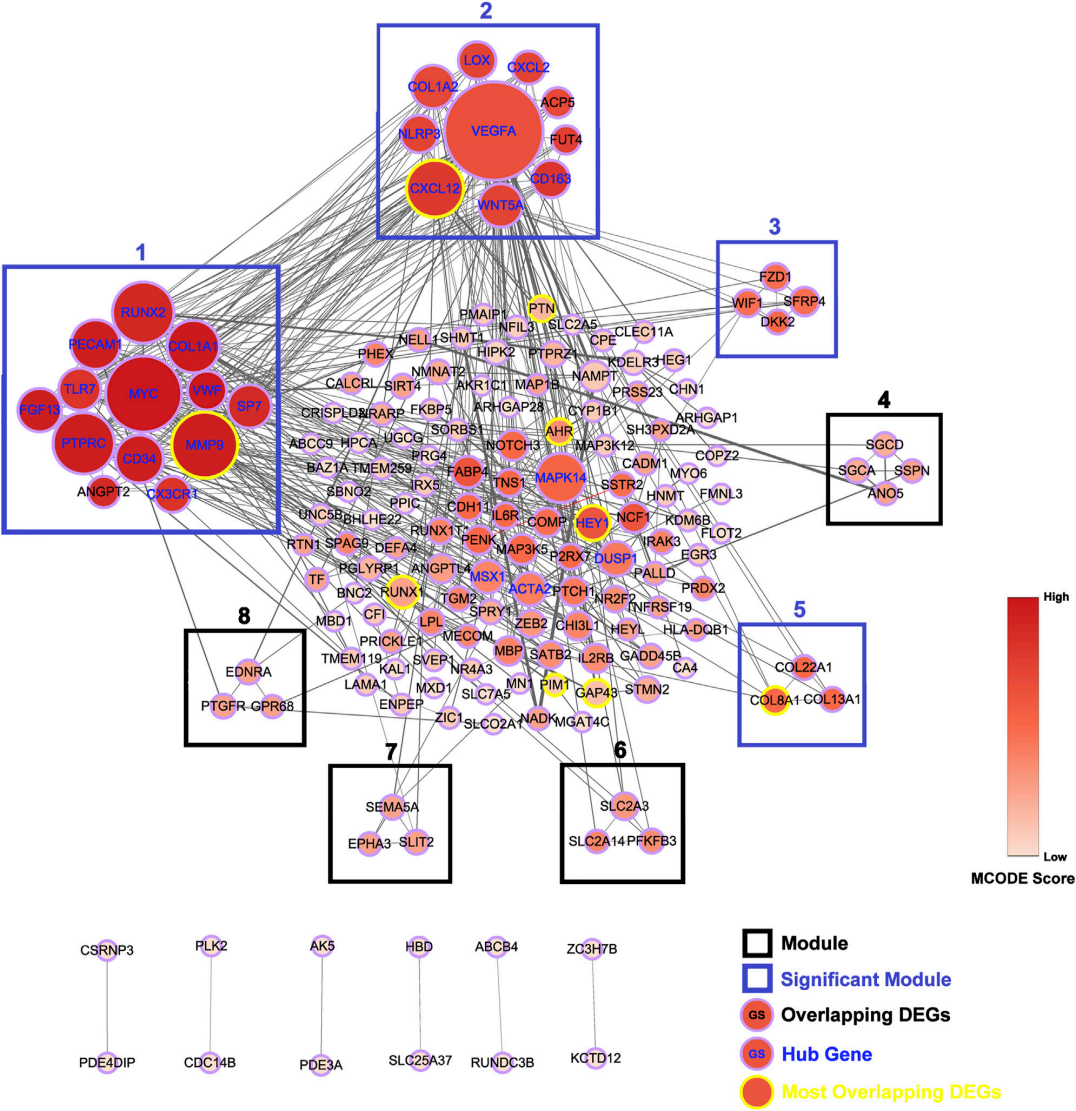
Construction of protein-protein interaction (PPI) network. PPI network for all the overlapping DEGs is constructed and followed by module analysis on Cytoscape platform. Genes with yellow border are most overlapping DEGs and genes with blue gene symbol are hub genes. Modules with blue border are significant modules. The size of circles reflects the degree of connectivity. The shades of color reflect MCODE score.

The GO enrichment analysis results were presented in Figure 3. For upregulated overlapping DEGs, the most enriched GO Molecular Functions were identified as “proteoglycan binding,” “extracellular matrix structural constituent,” “lipid binding,” “collagen binding,” and “Wnt-protein binding” (Figure 2A). The most enriched GO Biological Processes mainly included “blood vessel development,” “ossification,” “cell morphogenesis involved in differentiation,” “cellular response to growth factor stimulus,” “response to mechanical stimulus” and “extracellular structure organization.” In addition, the most enriched GO Cellular Components were “extracellular matrix,” “cell-cell junction,” “dystrophin-associated glycoprotein complex,” “filopodium,” and “distal axon,” etc. For downregulated overlapping DEGs, the most enriched GO Molecular Functions mainly included “glucose transmembrane transporter activity,” “protein homodimerization activity,” “signaling adaptor activity,” “transcription factor binding,” and “cytokine activity” (Figure 2B). The GO Biological Processes were enriched in “activation of protein kinase activity,” “glucose transmembrane transport,” “cellular response to leptin stimulus,” “SMAD protein signal transduction,” “response to interleukin-6,” and “response to toxic substance.” In addition, the most enriched GO Cellular Components were identified as “secretory granule lumen,” “apical plasma membrane,” “specific granule,” “adherent junction,” and “perinuclear region of cytoplasm.”

### KEGG Pathway Analysis

KEGG pathway analysis by Metascape indicated that total overlapping DEGs were enriched in 17 pathways including “Wnt signaling pathway,” “Fluid shear stress,” “Axon guidance,” “Nicotinate and nicotinamide metabolism,” “PI3K-Akt signaling pathway,” “HIF-1 signaling pathway,” “MAPK signaling pathway,” “Cytokine-cytokine receptor interaction,” “PPAR signaling pathway,” “NOD-like receptor signaling pathway,” and “TGF-beta signaling pathway,” etc. (Figure 2C). Enriched genes locating in corresponding pathways were summarized in Table 3. *MYC, VEGFA, IL2RB, MAPK14, IL6R, MMP9, HLA-DQB1,* and *CXCL12* were most enriched in these identified KEGG pathways.

**TABLE 3:** Enriched pathways and corresponding genes.

Pathway	−Log 10(P)	Enriched Genes
Wnt signaling pathway	3.882	FZD1, WNT5A, MYC, WIF1, SFRP4, DKK2, PRICKLE1
Transcriptional misregulation in cancer	3.427	RUNX1, MYC, RUNX1T1, NR4A3, RUNX2, TSPAN7, MMP9, IL2RB
Fluid shear stress and atherosclerosis	3.329	MMP9, MAPK14, MAP3K5, PECAM1, NCF1, DUSP1, VEGFA
PI3K-Akt signaling pathway	3.283	COL1A2, COL1A1, VWF, LAMA1, COMP, VEGFA, ANGPT2, IL6R, IL2RB, FGF13, MYC
Th17 cell differentiation	3.217	HLA-DQB1, MAPK14, IL2RB, IL6R, RUNX1, AHR
Axon guidance	2.797	PTCH1, UNC5B, WNT5A, SEMA5A, SLIT2, EPHA3, CXCL12
Nicotinate and nicotinamide metabolism	2.570	NADK, NAMPT, NMNAT2
HIF-1 signaling pathway	2.524	IL6R, PFKFB3, ANGPT2, VEGFA, TF, LOX
MAPK signaling pathway	2.462	GADD45B, MAP3K12, MAPK14, MAP3K5, MECOM, FGF13, MYC, DUSP1
Cytokine-cytokine receptor interaction	2.314	VEGFA, IL6R, CX3CR1, IL2RB, INHBB, TNFRSF19, CXCL12, CXCL2
PPAR signaling pathway	2.284	ANGPTL4, FABP4, LPL, SORBS1
Bladder cancer	2.142	MMP9, VEGFA, MYC
Rheumatoid arthritis	1.947	HLA-DQB1, VEGFA, ACP5, CXCL12
Cell adhesion molecules (CAMs)	1.885	CADM1, CD34, HLA-DQB1, PTPRC, PECAM1
Viral myocarditis	1.713	SGCD, SGCA, HLA-DQB1
NOD-like receptor signaling pathway	1.606	MAPK14, P2RX7, NAMPT, NLRP3, CXCL2
TGF-β signaling pathway	1.321	INHBB, BMP8B, MYC

FZD1, frizzled-1; WNT5A, Wnt-5a; MYC, c-myc; WIF1, Wnt inhibitory factor 1; SFRP4, secreted frizzled-related protein 4; DKK2, dickkopf-related protein 2; PRICKLE1, prickle planar cell polarity protein 1; RUNX1, runt-related transcription factor 1; RUNX1T1, RUNX1 partner transcriptional co-repressor 1; NR4A3, nuclear receptor subfamily 4, group A, member 3; RUNX2, runt-related transcription factor 2; TSPAN7, tetraspanin-7; MMP9, matrix metallopeptidase 9; IL2RB, interleukin-2 receptor subunit beta; MAPK14, mitogen-activated protein kinase 14; MAP3K5, mitogen-activated protein kinase 5; PECAM1, platelet endothelial cell adhesion molecule 1; NCF1, Neutrophil cytosol factor 1; DUSP1, dual specificity protein phosphatase 1; VEGFA, vascular endothelial growth factor A; COL1A2, collagen type I alpha 2 chain; COL1A1, collagen type I alpha 2 chain; VWF, Von Willebrand factor; LAMA1, laminin subunit alpha-1; COMP, cartilage oligomeric matrix protein; ANGPT2, angiopoietin 2; IL6R, interleukin 6 receptor; IL2RB, interleukin 2 receptor subunit beta; FGF13, fibroblast growth factor 13; HLA-DQB1, major histocompatibility complex, class II, DQ beta 1; AHR, aryl hydrocarbon receptor; PTCH1, patched 1; UNC5B, unc-5 netrin receptor B; SEMA5A, semaphorin 5A; SLIT2, slit guidance ligand 2; EPHA3, EPH receptor A3; CXCL12/SDF1, stromal cell-derived factor 1; NADK, NAD kinase; NAMPT, nicotinamide phosphoribosyltransferase; NMNAT2, nicotinamide nucleotide adenylyltransferase 2; PFKFB3, 6-phosphofructo-2-kinase/fructose-2,6-biphosphatase 3; TF, transferrin; GADD45B, growth arrest and DNA damage inducible beta; MAP3K12, mitogen-activated protein kinase kinase kinase 12; MECOM, MDS1 and EVI1 complex locus; CX3CR1, C-X3-C motif chemokine receptor 1; INHBB, inhibin subunit beta B; TNFRSF19, TNF receptor superfamily member 19; CXCL2, C-X-C motif chemokine ligand 2; ANGPTL4, angiopoietin like 4; FABP4, fatty acid binding protein 4; LPL, lipoprotein lipase; SORBS1, Sorbin and SH3 domain containing 1; ACP5, acid phosphatase 5; CADM1, cell adhesion molecule 1; PTPRC, protein tyrosine phosphatase receptor type C; SGCD, sarcoglycan delta; SGCA, sarcoglycan alpha; P2RX7, purinergic receptor P2X 7; NLRP3, NLR family Pyrin domain containing 3; BMP8B, bone morphogenetic protein 8b.

### Construction of the PPI Network

A total of 168 interactions were obtained with interaction scores>0.4 by using STRING database. The PPI network was then constructed and presented at Cytoscape platform (Figure 3). In addition, 25 hub genes were obtained and presented in Table 4. The top 10 hub genes included *VEGFA, MYC, MMP9, RUNX2, PTPRC, CXCL12, COL1A1, MAPK14, PECAM1,* and *CD34*.

**TABLE 4:** List of hub genes and corresponding connectivity degree.

Gene	Degree	Gene	Degree	Gene	Degree	Gene	Degree	Gene	Degree
VEGFA	54	CXCL12	26	COL1A2	17	VWF	14	DUSP1	11
MYC	39	COL1A1	23	WNT5A	17	NLRP3	13	HEY1	11
MMP9	32	MAPK14	21	SP7	17	LOX	13	CXCL2	10
RUNX2	29	PECAM1	21	FGF13	17	CD163	13	ACTA2	10
PTPRC	29	CD34	20	TLR7	16	CX3CR1	12	MSX1	10

VEGFA, vascular endothelial growth factor A; MYC, c-myc; MMP9, matrix metallopeptidase 9; RUNX2, runt-related transcription factor 2; PTPRC, protein tyrosine phosphatase receptor type C; CXCL12/SDF1, stromal cell-derived factor 1; COL1A1, collagen type I alpha 1 chain; MAPK14, mitogen-activated protein kinase 14; PECAM1, platelet endothelial cell adhesion molecule 1; COL1A2, collagen type I alpha 2 chain; WNT5A, Wnt-5a; SP7, Sp7 Transcription Factor; FGF13, fibroblast growth factor 13; TLR7, Toll-like receptor 7; VWF, Von Willebrand factor; NLRP3, NLR family Pyrin domain containing 3; LOX, lysyl oxidase; CX3CR1, C-X3-C motif chemokine receptor 1; DUSP1, dual specificity phosphatase 1; HEY1, Hes related family BHLH transcription factor with YRPW motif 1; CXCL2, C-X-C motif chemokine ligand 2; ACTA2, actin alpha 2; MSX1, Msh homeobox 1.

### Module Analysis

**FIGURE 4:**
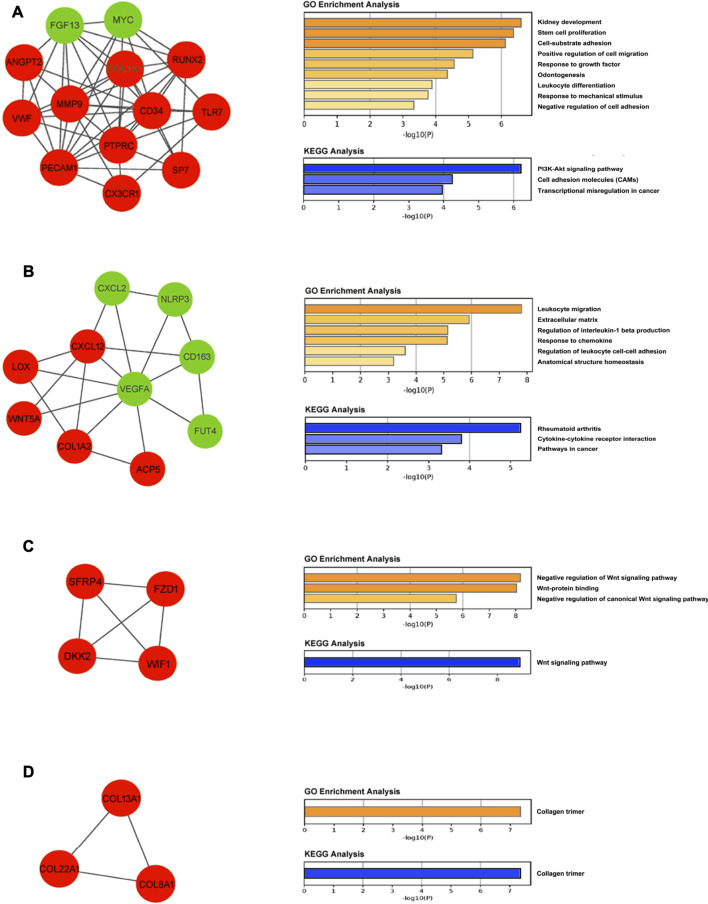
Module analysis. **(A)** Module one is comprised of 13 genes and enriched in PI3K-Akt signaling pathway and cell adhesion molecules (CAMs). **(B)** Module two is comprised of 10 genes and enriched in rheumatoid arthritis pathway and cytokine-cytokine receptor interaction pathway. **(C)** Module three is comprised of four genes and enriched in the Wnt signaling pathway. **(D)** Module five is comprised of three genes and enriched in collagen trimer pathway. Upregulated and downregulated genes are stained with red and green, respectively.

A total of eight modules and four significant modules (Module 1, 2, 3, and 5) were obtained through MCODE analysis (Figure 3). Among significant modules, Module 1 included a total of 13 genes, of which 11 were upregulated and two were downregulated. GO Enrichment analysis showed that Module one was enriched in nine functions such as “stem cell proliferation,” “response to growth factor,” and “response to mechanical stimulus,” etc. For pathway analysis, Module one was significantly enriched in pathways such as “PI3K-Akt signaling pathway” and “Cell adhesion molecules (CAMs)” (Figure 4A). Module two was composited of 10 genes, of which five were upregulated and five were downregulated. Module two was enriched in six functions such as “regulation of interleukin-1 beta production,” “response to chemokine,” “anatomical structure homeostasis,” etc. In addition, “Rheumatoid arthritis” and “Cytokine-cytokine receptor interaction” pathways were enriched by genes within Module 2 (Figure 4B). Module three included four genes and all of them were upregulated. Enrichment analysis showed that Module three was enriched in “Wnt-protein binding” and “Wnt signaling pathway” (Figure 4C). Module five included three genes, all of them were upregulated. Enrichment analysis suggested that Module five was enriched in “collagen trimer” pathway (Figure 4D).

### Construction of the miRNA-mRNA Network

**FIGURE 5:**
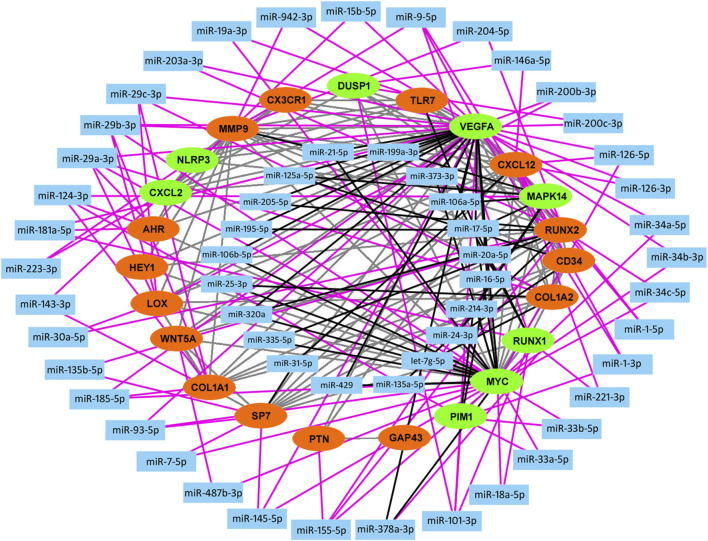
Construction of miRNA-mRNA network. A total of 23 knee OA-related key genes (red, upregulated; green, downregulated) are used in construction of miRNA-mRNA network backbone. Key miRNAs are then screened out by miRTarBase database and visualized in Cytoscape. Grey line, protein-protein interaction; Black line, interaction between key gene and key miRNA which belongs to inner circle. Pink line, interaction between key gene and key miRNA which belongs to outside circle.

The above identified most overlapping DEGs and hub genes were regarded as knee OA-related key genes. By constructing the PPI network of these key genes, we then screened out the experimentally validated key gene-related miRNAs targeting at above OA-related key genes by using miRTarBase (Table 5). As a result, a total of 57 key miRNAs were obtained and visualized in Figure 5. The top 10 key miRNAs included miR-29, miR-101, miR-17, miR-181a, miR-124, miR-1, miR-9, miR-21, miR-155, and miR-185. Key miRNAs were further compared with OA-related miRNAs that were obtained from the Human microRNA Disease Database (HMDD v3.2, http://www.cuilab.cn/hmdd) to check the reliability of our analyses and screen out miRNAs that lack researches so far (Huang et al., 2019).

**TABLE 5:** List of miRNAs targeting at knee OA-related key genes.

Gene	miRNAs
AHR	miR-124-3p, miR-181a-5p, miR-29a-3p
HEY1	miR-410-3p
CXCL12	miR-23a-3p, miR-31-5p, miR-886-3p, miR-126-5p, miR-126-3p, miR-146a-5p, miR-221-3p, miR-454-3p, miR-137, miR-1-5p, miR-1-3p, miR-448
MMP9	miR-451a, miR-491-5p, miR-338-3p, miR-204-5p, miR-21-5p, miR-9-5p, miR-211-5p, let-7e-5p, miR-133b, miR-29b-3p, miR-9-3p, miR-524-5p, miR-302a-5p, miR-132-3p, miR-15b-5p, miR-942-3p, miR-203a-5p, miR-133a-5p, miR-143-3p
RUNX1	miR-17-5p, miR-20a-5p, miR-106a-5p, miR-675-5p, miR-221-3p, miR-27b-3p, miR-18a-5p, miR-215-5p, miR-9-5p, miR-101-3p, miR-144-5p, miR-181a-5p, miR-378a-3p
GAP43	miR-363-3p
PTN	miR-155-5p
PIM1	miR-210-3p, miR-1-3p, miR-192-5p, miR-16-5p, miR-33a-5p, miR-33b-5p, miR-214-3p, miR-124-3p, miR-542-3p, miR-101-3p, miR-486-5p
VEGFA	miR-373-3p, miR-302d-3p, miR-126-3p, miR-147a, miR-134-5p, miR-140-5p, miR-29b-3p, miR-107, miR-16-5p, miR-93-5p, miR-17-5p, miR-150-5p, miR-195-5p, miR-15b-5p, miR-15a-5p, miR-520g-3p, miR-378a-3p, miR-330-3p, miR-383-5p, miR-125a-5p, miR-361-5p, miR-20a-5p, miR-20b-5p, miR-504-5p, miR-520h, miR-372-3p, miR-106a-5p, miR-106b-5p, miR-34a-5p, miR-205-5p, miR-34b-3p, miR-145-5p, miR-200b-3p, miR-200c-3p, miR-503-5p, miR-29c-3p, miR-9-5p, miR-133a-3p, miR-101-3p, miR-21-5p, miR-203a-3p, miR-29a-3p, miR-718, miR-185-5p, miR-199a-5p, miR-374b-5p, miR-1-3p, miR-125a-3p, miR-320a, miR-126-5p, miR-186-5p, miR-205-3p, miR-1-5p, miR-101-5p, miR-181a-5p, miR-942-3p, miR-206, miR-296-5p, miR-199a-3p, miR-16-1-3p, miR-429
MYC	miR-24-3p, let-7a-5p, let-7g-5p, miR-34a-5p, miR-98-5p, let-7c-5p, miR-26a-5p, miR-145-5p, miR-21-5p, miR-34b-5p, miR-34c-5p, miR-18a-5p, miR-17-5p, miR-20a-5p, miR-34b-3p, miR-378a-3p, miR-371a-3p, miR-373-3p, miR-33b-5p, miR-135a-5p, miR-449c-5p, miR-429, miR-335-5p, let-7f-5p, miR-320b, miR-744-5p, miR-320a, miR-148a-3p, miR-212-3p, miR-494-3p, miR-155-5p, miR-33a-5p, miR-449a, miR-487b-3p, miR-7-5p, miR-93-5p, miR-324-3p, miR-184, miR-126-5p, miR-25-3p, miR-92a-2-5p, miR-92a-1-5p, miR-19b-2-5p, miR-19b-1-5p, miR-19a-3p, miR-106b-5p, miR-130a-3p, miR-25-5p, miR-185-5p, miR-29a-3p, miR-561-3p, miR-34a-3p, miR-599, miR-29b-3p, miR-129-2-3p
RUNX2	miR-135b-5p, miR-155-5p, miR-335-5p, miR-203a-3p, miR-497-5p, miR-195-5p, miR-204-5p, miR-433-3p, miR-30d-5p, miR-30a-5p, miR-30b-5p, miR-218-5p, miR-23b-3p, miR-34c-5p, miR-30c-5p, miR-205-5p, miR-320a, miR-135a-5p, miR-222-3p, miR-221-5p, miR-628-3p, miR-103a-3p, miR-30a-3p, miR-376c-3p
COL1A1	miR-29b-3p, miR-29c-3p, miR-143-3p, miR-133a-3p, miR-124-3p, miR-29b-1-5p, miR-185-5p, miR-129-5p
WNT5A	miR-374a-5p, miR-487b-3p, miR-516a-3p, miR-154-5p, miR-154-3p
SP7	miR-135b-5p, miR-637, miR-7-5p, miR-93-5p, miR-31-5p, miR-145-5p
TLR7	miR-758-3p, miR-17-5p, miR-19a-3p
NLRP3	miR-223-3p
LOX	miR-29a-3p, miR-29b-3p, miR-29c-3p, miR-767-5p, miR-200b-3p, miR-30a-5p
CX3CR1	miR-296-3p
DUSP1	miR-101-3p, miR-200c-3p, miR-940, miR-146a-5p
CXCL2	miR-223-3p, miR-532-5p
MAPK14	miR-124-3p, miR-24-3p, miR-199a-3p, miR-200a-3p, miR-141-3p, miR-125b-5p, miR-214-3p, miR-155-5p, miR-17-5p, miR-106a-5p, miR-128-3p, miR-27a-3p, miR-125a-5p
CD34	miR-125a-5p, miR-9-5p, miR-24-3p, miR-377-3p
COL1A2	let-7g-5p, miR-29c-3p, miR-26b-5p, miR-25-3p, miR-29a-3p

### Clinical Validation

**FIGURE 6:**
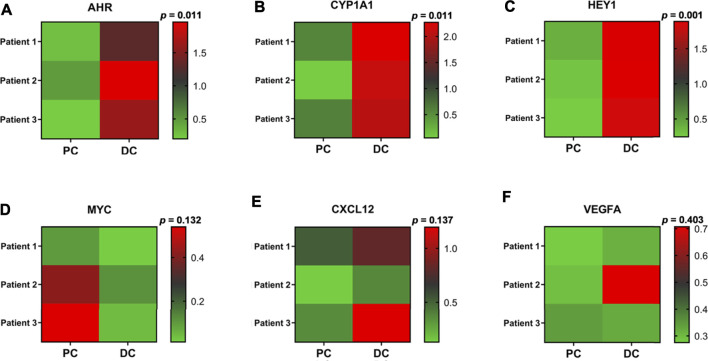
Clinical validation. **(A–F)** Heat maps of the relative expression of several identified knee OA-related key genes in osteoarthritic cartilage derived from knee OA patients. *p*, P-value; PC, preserved cartilage; DC, degenerated cartilage.

Quantitatively Real-time PCR assay (qRT-PCR) was performed to evaluate the relative expression of putative knee OA-related key genes in osteoarthritic cartilage specimens. As a result, significantly upregulated *AHR*, *CYP1A1*, and *HEY1* were observed in degenerated cartilage compared to the intact cartilage. Besides, *MYC* and *CXCL12* were also upregulated, while no significant difference was observed (Figures 6A–F).

## Discussion

The present study aims to screen out key DEGs, their relevant signaling pathways, and interactive miRNAs in human knee OA based on bioinformatic analysis. The gene expression profiles derived from different joint tissues are covered and followed by identifying overlapping DEGs. To the best of our knowledge, this is the first time that overlapping DEGs in knee OA are identified from four different OA joint tissues, including articular cartilage, synovial membrane, subchondral bone, and meniscus. Gene expression profiles are all obtained from GEO database.

After identification of DEGs in different joint tissues, overlapping DEGs are then identified *via* Venn Diagram. As a result, 236 overlapping DEGs are identified, of which 160 are upregulated and 76 are downregulated. Those DEGs that are differentially expressed in at least three joint tissues are identified as the most overlapping DEGs. As a result, a total of 13 most overlapping DEGs are identified in our study, include *AHR*, *HEY1*, *CXCL12*, *MMP9*, *COL8A1*, *GAP43*, *PTN*, *RUNX1*, *PIM1*, *OLFML2A*, *SLITRK6*, *RHBDL2*, and *ARL4C*, while no overlapping genes are differentially expressed in all four components. Among above identified most overlapping DEGs, *OLFML2A*, *SLITRK6*, *RHBDL2*, and *ARL4C* are excluded from our constructed PPI network, suggesting their relatively limited values and will not be discussed in the following context.

MMP9 is an enzyme implicated in the cartilage destruction and has been identified as a hallmark of OA previously, the same as other MMPs like MMP1, MMP3 and MMP13. Previous studies have reported the upregulation of MMP9 in OA cartilage, synovial membrane, subchondral bone, and synovial fluid (Yang et al., 2010; Kim et al., 2014). COL8A1, which maintains cartilage stability through participating in collagen synthesis, is also reported to be highly expressed in OA cartilage (Fang et al., 2019). CXCL12, also known as SDF-1, is a chemokine that plays important role in angiogenesis, bone metabolism, cartilage homeostasis, and pro-inflammatory responses (De Klerck et al., 2005; Garcia-Cuesta et al., 2019). Upregulation of CXCL12 in OA cartilage, subchondral bone, synoviocytes, and synovial fluid has been reported by previous studies (Chen et al., 2017b; Bragg et al., 2019). Furthermore, inhibition of CXCL12/CXCR4 signaling was shown to prevent subchondral bone loss and significantly attenuate cartilage degradation (Dong et al., 2016; Chen et al., 2017b). GAP43 is a nervous tissue-specific cytoplasmic protein related to nerve regeneration. Previous study showed that the expression of GAP43 in pain-related sensory innervation of dorsal-root ganglia (DRG) was upregulated during OA progression (Kawarai et al., 2018). Thus, the upregulation of GAP43 in joint tissues may reflect joint sensory innervation, which is closed related to nociceptive sense and osteophyte formation (Wu et al., 2002; Orita et al., 2011). Pleiotrophin (PTN) is an 18-kDa heparin-binding neurite outgrowth-promoting growth factor. Previous studies have reported the upregulation of PTN in OA cartilage, synovial membrane, and synovial fluid. Notably, PTN is initially abundant in fetal or juvenile cartilage and then becomes absent in mature cartilage. During early stages of OA, PTN becomes re-expressed again (Pufe et al., 2003; Mentlein 2007). Besides, PTN is also proven to facilitate chondrocyte proliferation (Pufe et al., 2007). Interestingly, GAP43 and PTN are two nerve growth-related genes, suggesting the important role of nerve innervation and axon guidance during OA development in the entire joint. Their specific role in OA development needs to be further investigated. PIM1 is an enzyme that play important roles in cell cycle progression, apoptosis, and transcriptional activation (Bachmann and Moroy, 2005). However, there is no study reporting their relationships so far.

The rest of most overlapping DEGs, including *HEY1*, *AHR*, *RUNX1*, and *HEY1*, are all transcription factors. HEY1 is a basic helix-loop-helix protein (bHLH) transcription factor that belongs to HES/HEY family. Besides, HEY1 is also a direct target of canonical Notch signaling. Previous studies indicated that inhibition of Notch1 exacerbated experimental OA, while increased levels or activity of Notch2 contributed to the progression of OA (Hosaka et al., 2013; Lin et al., 2016). In addition, HEY1 is not only transcriptionally induced by Notch ligands, but also induced by BMP/TGF-β axis to exert as a transcription repressor. Its functions on repressing osteogenic differentiation, neuronal differentiation, and pro-inflammatory production have been reported before (Weber et al., 2014). Hes1, another transcription factor that belongs to HES/HEY family like HEY1, has been reported to be upregulated in OA cartilage and accelerate cartilage destruction *via* promoting MMP3, a disintegrin and metalloproteinase with thrombospondin motifs 5 (ADAMTS5), and interleukin-6 (IL-6) transcription (Sugita et al., 2015). Nonetheless, the specific role of HEY1 in OA progression remain to be investigated. AHR is a bHLH transcription factor that plays important role in development, immune system, and toxic response. Previous studies have demonstrated that AHR signaling activation significantly alleviated progression of rheumatoid arthritis (RA) through repressing C-reactive protein (CRP), NLR family pyrin domain containing 3 (NLRP3), tumor necrosis factor-alpha (TNF-α), and IL-6 expression (Jin et al., 2011; Huai et al., 2014; Liang et al., 2019; Piper et al., 2019), and enhancing nuclear factor erythroid 2-related factor 2 (NRF2) and IL-10 expression in B cells, macrophages, or hepatocytes (Tsuji et al., 2012; Piper et al., 2019; Rosser et al., 2020). However, several reports also indicated that AHR signaling activation exacerbated RA inflammation through activating cytokine-mediated inflammatory signaling in primary human fibroblast-like synoviocytes (Adachi et al., 2013; Lahoti et al., 2013). Ogando J et al. reported that the AHR signaling pathway was significantly more active in OA synovial tissues than in RA synovial tissues (Ogando et al., 2016). Furthermore, compared to resting chondrocytes, significantly upregulated AHR was also observed in hypertrophic chondrocytes (Cedervall et al., 2015). Nevertheless, the specific effects of AHR on OA are still in debate and remain to be investigated. RUNX1 was reported to be significantly downregulated in OA cartilage compared with normal control. Furthermore, its anabolic effect on chondrocytes contributes to the maintenance of cartilage homeostasis during OA development and that has also been recognized as a disease-modifying target of OA (Yano et al., 2013; Aini et al., 2016; Yano et al., 2019). In addition, the anti-angiogenic effect of RUNX1 through repressing vascular endothelial growth factor (VEGF) expression has also been reported (Ter Elst et al., 2011).

According to KEGG pathway analysis based on overlapping DEGs, we observe that overlapping DEGs are enriched in “PI3K-Akt signaling pathway,” “Wnt signaling pathway,” “Fluid shear stress,” “Nicotinate and nicotinamide metabolism,” “HIF-1 signaling pathway,” “MAPK signaling pathway,” “Cytokine-cytokine receptor interaction,” “PPAR signaling pathway,” “NOD-like receptor signaling pathway,” “Axon guidance,” and “TGF-β signaling pathway.” The above pathways are well consistent with existing research findings. For example, PI3K-Akt signaling and MAPK signaling are closely involved in inflammatory response to induce the production of catabolic markers such as MMPs, Adamts, IL-1β, and TNF-α in OA (Herrero-Beaumont et al., 2019; Chow and Chin, 2020). Nicotinate and nicotinamide metabolism play important roles in redox reaction. So far, several studies have reported the important role of nicotinate and nicotinamide metabolism in OA development (Yang et al., 2015; Junker et al., 2017). In 2015, Yang et al. (2015) demonstrated that nicotinamide phosphoribosyltransferase (NAMPT), a rate-limiting enzyme in the Nicotinamide adenine dinucleotide (NAD+) salvage pathway, acted as a crucial catabolic regulator of osteoarthritic cartilage destruction. In addition, aberrantly activated Wnt signaling or TGF-β signaling contributes to cartilage degradation, osteophyte formation, and formation of subchondral bone marrow osteoid islets (Zhen et al., 2013; van der Kraan 2017). Therapies targeting Wnt signaling have been undergoing clinical trials (ClinicalTrials.gov Identifier: NCT03928184). HIF-1α and HIF-2α were also reported to exert anabolic and catabolic effects on chondrocytes, respectively (Zhang et al., 2015). PPAR signaling, in particular PPARγ, which is significantly downregulated in OA cartilage, has been demonstrated to maintain articular cartilage homeostasis *via* regulating the mTOR pathway (Vasheghani et al., 2015; Zhu et al., 2019). NOD-like receptor signaling, which mediates innate immunity and participates in regulating inflammatory and apoptotic responses, mainly includes two subfamilies, NODs and NLRPs (Platnich and Muruve, 2019). Both NOD-dependent pathway and NLRP-dependent inflammasome pathway were validated to mediate OA development under external stimulus (Jin et al., 2011; Xu et al., 2015). Fluid shear stress (FSS) was known as one of the pathogenic mechanisms of OA and has also been used in the construction of an osteoarthritic cell model for a long time (Yang et al., 2020). In addition to above well-validated OA related-pathways, axon guidance pathway is lack of research up to now. Only several *in vitro* studies reported the effects of an axon guidance molecule, Semaphorin 3A (Sema3A), on osteoarthritic chondrocytes (Okubo et al., 2011; Sumi et al., 2018).

Through constructing the PPI network and miRNA-mRNA network in Cytoscape, a total of 25 hub genes, four significant modules, and 57 key miRNAs are identified. Among hub genes, *MAPK14*, *IL2RB*, and *IL6R* are all involved in cytokine-mediated inflammatory response during OA (Boileau et al., 2005, Liang et al., 2018, Shkhyan et al., 2018). A genome-wide association (GWAS) study has identified a single nucleotide polymorphism (SNP) of *HLA-DQB1*, rs7775228, associating with knee OA in Asian population (Valdes and Spector, 2011). Furthermore, a small molecule gp130 modulator (RCGD 423) was proven to improve chondrocyte proliferation and inhibit cartilage degradation *via* upregulating transcription factor MYC and suppressing IL-6-mediated inflammatory response (Shkhyan et al., 2018). For growth factor VEGFA, it is closely related to angiogenesis and inflammatory response (Gao et al., 2013). To validate above computational results, we performed qRT-PCR to validate the relative expression of identified key genes in OA cartilage clinically. Our results show that *AHR* and its downstream target *CYP1A1*, *HEY1*, *MYC*, and *CXCL12* are indeed upregulated in degenerated cartilage compared to preserved cartilage (Figure 6).

According to module analysis, majority of hub genes are located in module one and module 2. In addition, *MMP9*, *CXCL12*, and *HEY1* are not only the most overlapping DEGs, but also hub genes. Module analysis shows that Modules 1, 2, 3, and 5 are significant modules with the MCODE score ≥4. For Module 1, all of the genes within Module 1, except *ANGPT2*, are hub genes. *MMP9* is located in Module 1. Enrichment analysis suggests that Module one plays important role in PI3K-Akt signaling pathway and cell adhesion molecules (CAMs) pathway. Regarding genes within Module 2, all of them are hub genes except *ACP5* and *FUT4*. Genes in Module two play important roles in rheumatoid arthritis and cytokine-cytokine receptor interaction pathways. Furthermore, *CXCL12* is located in Module 2. Module three is a group of genes associated with Wnt signaling pathway. No most overlapping DEGs or hub genes locate in Module 3. Module five includes *COL8A1*, *COL13A1*, and *COL22A1*, all of which are associated with collagen trimer pathway and have been demonstrated to be aberrantly expressed in degraded cartilage (Karlsson et al., 2010; Feng et al., 2019). A recent study reported that Lgr5+/Col22a1+ stem cells play important roles in differentiation toward articular chondrocytes and Col22a1-expressing cartilage superficial layer contributed to repair of cartilage defect (Feng et al., 2019).

Collectively, based on above identified overlapping DEGs, hub genes, pathways, and functional modules, the present study depicted the potential OA mechanisms covering the entire knee joint. We believe that our identified knee OA-related key genes, such as *AHR*, *HEY1*, *MYC*, *GAP43*, and *PTN*, and their relevant signaling pathways, including AHR signaling, Notch signaling and TGF-β signaling, C-MYC signaling, and axon guidance pathway may play important roles in knee OA development. Our predicted genes are worthy of being explored as novel targets of DMOADs in the future.

By comparing key miRNAs with OA-related miRNAs included in HMDD v3.2 database, 41 out of 57 miRNAs (about 71.9%) have been reported to be associated with OA, suggesting the considerable reliability of our miRNA-mRNA interactome predictions. For example, miR-29 family, including miR-29a, miR-29b, and miR-29c, was differentially expressed in OA cartilage and negatively regulated Smad, NF-κB, and canonical Wnt signaling (Le et al., 2016). In addition, upregulation of miR-101 significantly facilitated cartilage degradation and chondrocyte apoptosis (Dai et al., 2015; Lü et al., 2020). For miR-181a, Akihiro Nakamura et al. demonstrated that intra-articular injections of locked nucleic acid (LNA) miR-181a antisense oligonucleotides (ASO) significantly attenuated cartilage destruction in facet and knee joints *in vivo* (Nakamura et al., 2019). Another study reported that miR-9 promoted IL-6 expression and exacerbated cartilage degradation by targeting MCPIP1 expression (Makki et al., 2015). In 2019, a hydrogel-based drug delivery system equipped with locked nucleic acid (LNA) miR-221 inhibitor was constructed and enhanced cartilage regeneration significantly (Lolli et al., 2019). miR-199a* was also shown to inhibit IL-1β-induced Cyclooxygenase 2 (COX-2) expression as a direct regulator (Akhtar and Haqqi, 2012). Notably, a RNA sequencing-based miRNA-mRNA interactome study which confirmed a OA-specific miRNAs array showed considerable overlap with our identified key miRNAs as well, including miR-143, miR-155, let-7g, miR-7, miR-15, miR-101, miR-21, miR-19a, miR-16, miR-30a, miR-29, miR-1, miR-133a-3p, miR-20a, miR-320a, miR-31, miR-93, miR-221, and miR-335 (Coutinho de Almeida et al., 2019). Among our identified top 10 key miRNAs, in addition to those that have been reported before, miR-17, miR-21, miR-155, miR-185, and miR-1 are still lack of research and remain to be investigated in the future.

The present study also has several limitations. For example, although we originally intended to include as many data sets as possible, only four expression profiles met the criteria and were included into the data analysis. In addition, due to difficulties in obtaining appropriate specimens, only OA articular cartilage specimens were used to carry out clinical validation experiments. The relative expression of our identified knee OA-related key genes in other joint components need to be further validated.

In conclusion, the present study provides a comprehensive bioinformatics analysis of key genes, signaling pathways, and miRNAs in different joint tissues of knee OA patients. A total of 35 knee OA-related key genes and 57 key miRNAs were identified. Among them, key genes such as *AHR*, *HEY1*, *MYC*, *GAP43*, and *PTN*, and key miRNAs such as miR-17, miR-21, miR-155, miR-185, and miR-1 are lack of research so far. For key genes identified in the present study, their downstream mechanisms and specific effects on the different joint components also need to be explored, respectively. Through enrichment analysis, a number of OA-related pathways were identified, including PI3K-Akt signaling, Wnt signaling, fluid shear stress, nicotinate and nicotinamide metabolism, HIF-1 signaling, MAPK signaling, cytokine-cytokine receptor interaction, PPAR signaling, NOD-like receptor signaling, TGF-β signaling, and axon guidance pathways. Among them, many pathways have been well investigated and even under clinical trials, except for axon guidance pathway which is implicated in nerve innervation and axon guidance while still lack of research so far. Our study provides insight for the first time in identification of potential therapeutic targets of knee OA by comprehensively analyzing the overlapping genes differentially expressed in multiple joint components based on bioinformatics.

## Data Availability

The original contributions presented in the study are included in the article/supplementary material, further inquiries can be directed to the corresponding authors.

## References

[B1] Adachi M. Okamoto S. Chujyo S. Arakawa T. Yokoyama M. Yamada K. (2013). Cigarette Smoke Condensate Extracts Induce IL-1-Beta Production From Rheumatoid Arthritis Patient-Derived Synoviocytes, but Not Osteoarthritis Patient-Derived Synoviocytes, Through Aryl Hydrocarbon Receptor-Dependent NF-Kappa-B Activation and Novel NF-Kappa-B Sites. J. Interferon Cytokine Res. 33 (6), 297–307. 10.1089/jir.2012.0107 23452206

[B2] Aini H. Itaka K. Fujisawa A. Uchida H. Uchida S. Fukushima S. (2016). Messenger RNA Delivery of a Cartilage-Anabolic Transcription Factor as a Disease-Modifying Strategy for Osteoarthritis Treatment. Sci. Rep. 6, 18743. 10.1038/srep18743 26728350 PMC4700530

[B3] Akhtar N. Haqqi T. M. (2012). MicroRNA-199a* Regulates the Expression of Cyclooxygenase-2 in Human Chondrocytes. Ann. Rheum. Dis. 71 (6), 1073–1080. 10.1136/annrheumdis-2011-200519 22294637 PMC4509731

[B4] Bachmann M. Möröy T. (2005). The Serine/threonine Kinase Pim-1. Int. J. Biochem. Cel Biol. 37 (4), 726–730. 10.1016/j.biocel.2004.11.005 15694833

[B5] Boileau C. Pelletier J. P. Tardif G. Fahmi H. Laufer S. Lavigne M. (2005). The Regulation of Human MMP-13 by Licofelone, an Inhibitor of Cyclo-Oxygenases and 5-lipoxygenase, in Human Osteoarthritic Chondrocytes Is Mediated by the Inhibition of the P38 MAP Kinase Signalling Pathway. Ann. Rheum. Dis. 64 (6), 891–898. 10.1136/ard.2004.026906 15498796 PMC1755518

[B6] Bragg R. Gilbert W. Elmansi A. M. Isales C. M. Hamrick M. W. Hill W. D. (2019). Stromal Cell-Derived Factor-1 as a Potential Therapeutic Target for Osteoarthritis and Rheumatoid Arthritis. Ther. Adv. Chronic Dis. 10, 2040622319882531. 10.1177/2040622319882531 31695863 PMC6820172

[B7] Brophy R. H. Zhang B. Cai L. Wright R. W. Sandell L. J. Rai M. F. (2018). Transcriptome Comparison of Meniscus from Patients With and Without Osteoarthritis. Osteoarthritis Cartilage. 26 (3), 422–432. 10.1016/j.joca.2017.12.004 29258882 PMC6007850

[B8] Cedervall T. Lind P. M. Sävendahl L. (2015). Expression of the Aryl Hydrocarbon Receptor in Growth Plate Cartilage and the Impact of its Local Modulation on Longitudinal Bone Growth. Int. J. Mol. Sci. 16 (4), 8059–8069. 10.3390/ijms16048059 25867478 PMC4425067

[B9] Chang L. Liu A. Xu J. Xu X. Dai J. Wu R. (2021). TDP-43 Maintains Chondrocyte Homeostasis and Alleviates Cartilage Degradation in Osteoarthritis. Osteoarthritis Cartilage. 29 (7), 1036–1047. 10.1016/j.joca.2021.03.015 33781898

[B10] Chen D. Shen J. Zhao W. Wang T. Han L. Hamilton J. L. (2017a). Osteoarthritis: Toward a Comprehensive Understanding of Pathological Mechanism. Bone Res. 5, 16044. 10.1038/boneres.2016.44 28149655 PMC5240031

[B11] Chen Y. Lin S. Sun Y. Guo J. Lu Y. Suen C. W. (2017b). Attenuation of Subchondral Bone Abnormal Changes in Osteoarthritis by Inhibition of SDF-1 Signaling. Osteoarthritis Cartilage. 25 (6), 986–994. 10.1016/j.joca.2017.01.008 28131784

[B12] Choi W. S. Yang J. I. Kim W. Kim H. E. Kim S. K. Won Y. (2019). Critical Role for Arginase II in Osteoarthritis Pathogenesis. Ann. Rheum. Dis. 78 (3), 421–428. 10.1136/annrheumdis-2018-214282 30610061 PMC6390026

[B13] Chou C. H. Wu C. C. Song I. W. Chuang H. P. Lu L. S. Chang J. H. (2013). Genome-Wide Expression Profiles of Subchondral Bone in Osteoarthritis. Arthritis Res. Ther. 15 (R190), R190. 10.1186/ar4380 24229462 PMC3979015

[B14] Chow Y. Y. Chin K. Y. (2020). The Role of Inflammation in the Pathogenesis of Osteoarthritis. Mediators Inflamm. 2020, 8293921. 10.1155/2020/8293921 32189997 PMC7072120

[B15] Coutinho de Almeida R. Ramos Y. F. M. Mahfouz A. den Hollander W. Lakenberg N. Houtman E. (2019). RNA Sequencing Data Integration Reveals an miRNA Interactome of Osteoarthritis Cartilage. Ann. Rheum. Dis. 78 (2), 270–277. 10.1136/annrheumdis-2018-213882 30504444 PMC6352405

[B16] Dai L. Zhang X. Hu X. Liu Q. Man Z. Huang H. (2015). Silencing of miR-101 Prevents Cartilage Degradation by Regulating Extracellular Matrix-Related Genes in a Rat Model of Osteoarthritis. Mol. Ther. 23 (8), 1331–1340. 10.1038/mt.2015.61 25921548 PMC4817865

[B17] De Klerck B. Geboes L. Hatse S. Kelchtermans H. Meyvis Y. Vermeire K. (2005). Pro-Inflammatory Properties of Stromal Cell-Derived Factor-1 (CXCL12) in Collagen-Induced Arthritis. Arthritis Res. Ther. 7 (6), R1208–R1220. 10.1186/ar1806 16277673 PMC1297565

[B18] Dong Y. Liu H. Zhang X. Xu F. Qin L. Cheng P. (2016). Inhibition of SDF-1α/CXCR4 Signalling in Subchondral Bone Attenuates Post-Traumatic Osteoarthritis. Int. J. Mol. Sci. 17 (6), 943. 10.3390/ijms17060943 PMC4926476 27322244

[B19] Fang Y. Wang P. Xia L. Bai S. Shen Y. Li Q. (2019). Aberrantly Hydroxymethylated Differentially Expressed Genes and the Associated Protein Pathways in Osteoarthritis. PeerJ. 7, e6425. 10.7717/peerj.6425 30828485 PMC6394344

[B20] Feng C. Chan W. C. W. Lam Y. Wang X. Chen P. Niu B. (2019). Lgr5 and Col22a1 Mark Progenitor Cells in the Lineage Toward Juvenile Articular Chondrocytes. Stem Cel Rep. 13 (4), 713–729. 10.1016/j.stemcr.2019.08.006 PMC6829785 31522976

[B21] Gao W. Sweeney C. Walsh C. Rooney P. McCormick J. Veale D. J. (2013). Notch Signalling Pathways Mediate Synovial Angiogenesis in Response to Vascular Endothelial Growth Factor and Angiopoietin 2. Ann. Rheum. Dis. 72 (6), 1080–1088. 10.1136/annrheumdis-2012-201978 23161900 PMC3664379

[B22] García-Cuesta E. M. Santiago C. A. Vallejo-Díaz J. Juarranz Y. Rodríguez-Frade J. M. Mellado M. (2019). The Role of the CXCL12/CXCR4/ACKR3 Axis in Autoimmune Diseases. Front. Endocrinol. (Lausanne). 10, 585. 10.3389/fendo.2019.00585 31507535 PMC6718456

[B23] Herrero-Beaumont G. Pérez-Baos S. Sánchez-Pernaute O. Roman-Blas J. A. Lamuedra A. Largo R. (2019). Targeting Chronic Innate Inflammatory Pathways, the Main Road to Prevention of Osteoarthritis Progression. Biochem. Pharmacol. 165, 24–32. 10.1016/j.bcp.2019.02.030 30825432

[B24] Hosaka Y. Saito T. Sugita S. Hikata T. Kobayashi H. Fukai A. (2013). Notch Signaling in Chondrocytes Modulates Endochondral Ossification and Osteoarthritis Development. Proc. Natl. Acad. Sci. U S A. 110 (5), 1875–1880. 10.1073/pnas.1207458110 23319657 PMC3562777

[B25] Huai W. Zhao R. Song H. Zhao J. Zhang L. Zhang L. (2014). Aryl Hydrocarbon Receptor Negatively Regulates NLRP3 Inflammasome Activity by Inhibiting NLRP3 Transcription. Nat. Commun. 5, 4738. 10.1038/ncomms5738 25141024

[B26] Huang H. Y. Lin Y. C. Li J. Huang K. Y. Shrestha S. Hong H. C. (2020). miRTarBase 2020: Updates to the Experimentally Validated microRNA-Target Interaction Database. Nucleic Acids Res. 48 (D1), D148–D154. 10.1093/nar/gkz896 31647101 PMC7145596

[B27] Huang Z. Shi J. Gao Y. Cui C. Zhang S. Li J. (2019). HMDD v3.0: a Database for Experimentally Supported Human microRNA-Disease Associations. Nucleic Acids Res. 47 (D1), D1013–D1017. 10.1093/nar/gky1010 30364956 PMC6323994

[B28] Huber R. Hummert C. Gausmann U. Pohlers D. Koczan D. Guthke R. (2008). Identification of Intra-Group, Inter-Individual, and Gene-Specific Variances in mRNA Expression Profiles in the Rheumatoid Arthritis Synovial Membrane. Arthritis Res. Ther. 10 (4), R98. 10.1186/ar2485 18721452 PMC2575612

[B29] Jin C. Frayssinet P. Pelker R. Cwirka D. Hu B. Vignery A. (2011). NLRP3 Inflammasome Plays a Critical Role in the Pathogenesis of Hydroxyapatite-Associated Arthropathy. Proc. Natl. Acad. Sci. U S A. 108 (36), 14867–14872. 10.1073/pnas.1111101108 21856950 PMC3169126

[B30] Junker S. Frommer K. W. Krumbholz G. Tsiklauri L. Gerstberger R. Rehart S. (2017). Expression of Adipokines in Osteoarthritis Osteophytes and Their Effect on Osteoblasts. Matrix Biol. 62, 75–91. 10.1016/j.matbio.2016.11.005 27884778

[B31] Kang Y. J. Yoo J. I. Baek K. W. (2021). Differential Gene Expression Profile by RNA Sequencing Study of Elderly Osteoporotic Hip Fracture Patients with Sarcopenia. J. Orthop. Translat. 29, 10–18. 10.1016/j.jot.2021.04.009 34036042 PMC8138673

[B32] Karlsson C. Dehne T. Lindahl A. Brittberg M. Pruss A. Sittinger M. (2010). Genome-Wide Expression Profiling Reveals New Candidate Genes Associated with Osteoarthritis. Osteoarthritis Cartilage. 18 (4), 581–592. 10.1016/j.joca.2009.12.002 20060954

[B33] Kawarai Y. Orita S. Nakamura J. Miyamoto S. Suzuki M. Inage K. (2018). Changes in Proinflammatory Cytokines, Neuropeptides, and Microglia in an Animal Model of Monosodium Iodoacetate-Induced Hip Osteoarthritis. J. Orthop. Res. 36 (11), 2978–2986. 10.1002/jor.24065 29888808

[B34] Kim J. H. Jeon J. Shin M. Won Y. Lee M. Kwak J. S. (2014). Regulation of the Catabolic Cascade in Osteoarthritis by the Zinc-ZIP8-MTF1 Axis. Cell. 156 (4), 730–743. 10.1016/j.cell.2014.01.007 24529376

[B35] Klinger P. Beyer C. Ekici A. B. Carl H. D. Schett G. Swoboda B. (2013). The Transient Chondrocyte Phenotype in Human Osteophytic Cartilage: A Role of Pigment Epithelium-Derived Factor? Cartilage. 4 (3), 249–255. 10.1177/1947603513480809 26069671 PMC4297088

[B36] Kung L. H. W. Ravi V. Rowley L. Angelucci C. Fosang A. J. Bell K. M. (2018). Cartilage MicroRNA Dysregulation during the Onset and Progression of Mouse Osteoarthritis Is Independent of Aggrecanolysis and Overlaps with Candidates From End-Stage Human Disease. Arthritis Rheumatol. 70 (3), 383–395. 10.1002/art.40378 29145712

[B37] Kuttapitiya A. Assi L. Laing K. Hing C. Mitchell P. Whitley G. (2017). Microarray Analysis of Bone Marrow Lesions in Osteoarthritis Demonstrates Upregulation of Genes Implicated in Osteochondral Turnover, Neurogenesis and Inflammation. Ann. Rheum. Dis. 76 (10), 1764–1773. 10.1136/annrheumdis-2017-211396 28705915 PMC5629942

[B38] Lahoti T. S. John K. Hughes J. M. Kusnadi A. Murray I. A. Krishnegowda G. (2013). Aryl Hydrocarbon Receptor Antagonism Mitigates Cytokine-Mediated Inflammatory Signalling in Primary Human Fibroblast-Like Synoviocytes. Ann. Rheum. Dis. 72 (10), 1708–1716. 10.1136/annrheumdis-2012-202639 23349129 PMC4041386

[B39] Latourte A. Kloppenburg M. Richette P. (2020). Emerging Pharmaceutical Therapies for Osteoarthritis. Nat. Rev. Rheumatol. 16 (12), 673–688. 10.1038/s41584-020-00518-6 33122845

[B40] Le L. T. Swingler T. E. Crowe N. Vincent T. L. Barter M. J. Donell S. T. (2016). The MicroRNA-29 Family in Cartilage Homeostasis and Osteoarthritis. J. Mol. Med. (Berl). 94 (5), 583–596. 10.1007/s00109-015-1374-z 26687115 PMC4856728

[B41] Li Y. Pan D. Liu S. Xing X. Zhou H. Zhang B. (2021). Identification of Circ-Fam169a Sponges miR-583 Involved in the Regulation of Intervertebral Disc Degeneration. J. Orthop. Translat. 26, 121–131. 10.1016/j.jot.2020.07.007 33437631 PMC7773979

[B42] Liang C. Li J. Lu C. Xie D. Liu J. Zhong C. (2019). HIF1α Inhibition Facilitates Leflunomide-AHR-CRP Signaling to Attenuate Bone Erosion in CRP-Aberrant Rheumatoid Arthritis. Nat. Commun. 10 (1), 4579. 10.1038/s41467-019-12163-z 31594926 PMC6783548

[B43] Liang Y. Chen S. Yang Y. Lan C. Zhang G. Ji Z. (2018). Vasoactive Intestinal Peptide Alleviates Osteoarthritis Effectively via Inhibiting NF-Κb Signaling Pathway. J. Biomed. Sci. 25 (1), 25. 10.1186/s12929-018-0410-z 29540226 PMC5851098

[B44] Lin N. Y. Distler A. Beyer C. Philipi-Schöbinger A. Breda S. Dees C. (2016). Inhibition of Notch1 Promotes Hedgehog Signalling in a HES1-Dependent Manner in Chondrocytes and Exacerbates Experimental Osteoarthritis. Ann. Rheum. Dis. 75 (11), 2037–2044. 10.1136/annrheumdis-2015-208420 26851274

[B45] Lolli A. Sivasubramaniyan K. Vainieri M. L. Oieni J. Kops N. Yayon A. (2019). Hydrogel-Based Delivery of antimiR-221 Enhances Cartilage Regeneration by Endogenous Cells. J. Control. Release. 309, 220–230. 10.1016/j.jconrel.2019.07.040 31369767

[B46] Lü G. Li L. Wang B. Kuang L. (2020). LINC00623/miR-101/HRAS Axis Modulates IL-1β-Mediated ECM Degradation, Apoptosis and Senescence of Osteoarthritis Chondrocytes. Aging. 12 (4), 3218–3237. 10.18632/aging.102801 32062610 PMC7066905

[B47] Makki M. S. Haseeb A. Haqqi T. M. (2015). MicroRNA-9 Promotion of Interleukin-6 Expression by Inhibiting Monocyte Chemoattractant Protein-Induced Protein 1 Expression in Interleukin-1β-Stimulated Human Chondrocytes. Arthritis Rheumatol. 67 (8), 2117–2128. 10.1002/art.39173 25917063 PMC4519390

[B48] Mentlein R. (2007). Targeting Pleiotropin to Treat Osteoarthritis. Expert Opin. Ther. Targets. 11 (7), 861–867. 10.1517/14728222.11.7.861 17614755

[B49] Nakamura A. Rampersaud Y. R. Nakamura S. Sharma A. Zeng F. Rossomacha E. (2019). MicroRNA-181a-5p Antisense Oligonucleotides Attenuate Osteoarthritis in Facet and Knee Joints. Ann. Rheum. Dis. 78 (1), 111–121. 10.1136/annrheumdis-2018-213629 30287418

[B50] Ogando J. Tardáguila M. Díaz-Alderete A. Usategui A. Miranda-Ramos V. Martínez-Herrera D. J. (2016). Notch-Regulated miR-223 Targets the Aryl Hydrocarbon Receptor Pathway and Increases Cytokine Production in Macrophages From Rheumatoid Arthritis Patients. Sci. Rep. 6, 20223. 10.1038/srep20223 26838552 PMC4738320

[B51] Okubo M. Kimura T. Fujita Y. Mochizuki S. Niki Y. Enomoto H. (2011). Semaphorin 3A Is Expressed in Human Osteoarthritic Cartilage and Antagonizes Vascular Endothelial Growth Factor 165-promoted Chondrocyte Migration: an Implication for Chondrocyte Cloning. Arthritis Rheum. 63 (10), 3000–3009. 10.1002/art.30482 21953086

[B52] Orita S. Ishikawa T. Miyagi M. Ochiai N. Inoue G. Eguchi Y. (2011). Pain-Related Sensory Innervation in Monoiodoacetate-Induced Osteoarthritis in Rat Knees That Gradually Develops Neuronal Injury in Addition to Inflammatory Pain. BMC Musculoskelet. Disord. 12, 134. 10.1186/1471-2474-12-134 21679434 PMC3142251

[B53] Park R. Ji J. D. (2016). Unique Gene Expression Profile in Osteoarthritis Synovium Compared With Cartilage: Analysis of Publicly Accessible Microarray Datasets. Rheumatol. Int. 36 (6), 819–827. 10.1007/s00296-016-3451-1 26942917

[B54] Piper C. J. M. Rosser E. C. Oleinika K. Nistala K. Krausgruber T. Rendeiro A. F. (2019). Aryl Hydrocarbon Receptor Contributes to the Transcriptional Program of IL-10-Producing Regulatory B Cells. Cell Rep. 29 (7), 1878–e7. 10.1016/j.celrep.2019.10.018 31722204 PMC6856759

[B55] Platnich J. M. Muruve D. A. (2019). NOD-Like Receptors and Inflammasomes: A Review of Their Canonical and Non-Canonical Signaling Pathways. Arch. Biochem. Biophys. 670, 4–14. 10.1016/j.abb.2019.02.008 30772258

[B56] Pufe T. Bartscher M. Petersen W. Tillmann B. Mentlein R. (2003). Pleiotrophin, an Embryonic Differentiation and Growth Factor, Is Expressed in Osteoarthritis. Osteoarthritis Cartilage. 11 (4), 260–264. 10.1016/s1063-4584(02)00385-0 12681952

[B57] Pufe T. Groth G. Goldring M. B. Tillmann B. Mentlein R. (2007). Effects of Pleiotrophin, a Heparin-Binding Growth Factor, on Human Primary and Immortalized Chondrocytes. Osteoarthritis Cartilage. 15 (2), 155–162. 10.1016/j.joca.2006.07.005 16949312

[B58] Rosser E. C. Piper C. J. M. Matei D. E. Blair P. A. Rendeiro A. F. Orford M. (2020). Microbiota-Derived Metabolites Suppress Arthritis by Amplifying Aryl-Hydrocarbon Receptor Activation in Regulatory B Cells. Cell Metab. 31 (4), 837–e10. 10.1016/j.cmet.2020.03.003 32213346 PMC7156916

[B59] Sandell L. J. (2012). Etiology of Osteoarthritis: Genetics and Synovial Joint Development. Nat. Rev. Rheumatol. 8 (2), 77–89. 10.1038/nrrheum.2011.199 22231237

[B60] Shannon P. Markiel A. Ozier O. Baliga N. S. Wang J. T. Ramage D. (2003). Cytoscape: a Software Environment for Integrated Models of Biomolecular Interaction Networks. Genome Res. 13 (11), 2498–2504. 10.1101/gr.1239303 14597658 PMC403769

[B61] Shkhyan R. Van Handel B. Bogdanov J. Lee S. Yu Y. Scheinberg M. (2018). Drug-Induced Modulation of Gp130 Signalling Prevents Articular Cartilage Degeneration and Promotes Repair. Ann. Rheum. Dis. 77 (5), 760–769. 10.1136/annrheumdis-2017-212037 29436471 PMC8444286

[B62] Soul J. Dunn S. L. Anand S. Serracino-Inglott F. Schwartz J. M. Boot-Handford R. P. (2018). Stratification of Knee Osteoarthritis: Two Major Patient Subgroups Identified by Genome-Wide Expression Analysis of Articular Cartilage. Ann. Rheum. Dis. 77 (3), 423. 10.1136/annrheumdis-2017-212603 29273645 PMC5867416

[B63] Styrkarsdottir U. Lund S. H. Thorleifsson G. Zink F. Stefansson O. A. Sigurdsson J. K. (2018). Meta-analysis of Icelandic and UK Data Sets Identifies Missense Variants in SMO, IL11, COL11A1 and 13 More New Loci Associated With Osteoarthritis. Nat. Genet. 50 (12), 1681–1687. 10.1038/s41588-018-0247-0 30374069

[B64] Sugita S. Hosaka Y. Okada K. Mori D. Yano F. Kobayashi H. (2015). Transcription Factor Hes1 Modulates Osteoarthritis Development in Cooperation With Calcium/Calmodulin-Dependent Protein Kinase 2. Proc. Natl. Acad. Sci. U S A. 112 (10), 3080–3085. 10.1073/pnas.1419699112 25733872 PMC4364241

[B65] Sumi C. Hirose N. Yanoshita M. Takano M. Nishiyama S. Okamoto Y. (2018). Semaphorin 3A Inhibits Inflammation in Chondrocytes under Excessive Mechanical Stress. Mediators Inflamm. 2018, 5703651. 10.1155/2018/5703651 29849491 PMC5911320

[B66] Szklarczyk D. Gable A. L. Nastou K. C. Lyon D. Kirsch R. Pyysalo S. (2021). The STRING Database in 2021: Customizable Protein-Protein Networks, and Functional Characterization of User-Uploaded Gene/measurement Sets. Nucleic Acids Res. 49 (D1), D605–D612. 10.1093/nar/gkaa1074 33237311 PMC7779004

[B67] Ter Elst A. Ma B. Scherpen F. J. de Jonge H. J. Douwes J. Wierenga A. T. (2011). Repression of Vascular Endothelial Growth Factor Expression by the Runt-Related Transcription Factor 1 in Acute Myeloid Leukemia. Cancer Res. 71 (7), 2761–2771. 10.1158/0008-5472.CAN-10-0402 21447743

[B68] Tsuji G. Takahara M. Uchi H. Matsuda T. Chiba T. Takeuchi S. (2012). Identification of Ketoconazole as an AhR-Nrf2 Activator in Cultured Human Keratinocytes: the Basis of its Anti-inflammatory Effect. J. Invest. Dermatol. 132 (1), 59–68. 10.1038/jid.2011.194 21753779

[B69] Tuerlings M. van Hoolwerff M. Houtman E. Suchiman E. H. E. D. Lakenberg N. Mei H. (2021). RNA Sequencing Reveals Interacting Key Determinants of Osteoarthritis Acting in Subchondral Bone and Articular Cartilage: Identification of IL11 and CHADL as Attractive Treatment Targets. Arthritis Rheumatol. 73 (5), 789–799. 10.1002/art.41600 33258547 PMC8252798

[B70] Umeno J. Matsumoto T. Fuyuno Y. Esaki M. Torisu T. (2021). SLCO2A1 Gene Is the Causal Gene for Both Primary Hypertrophic Osteoarthropathy and Hereditary Chronic Enteropathy. J. Orthop. Translat. 28, 10–11. 10.1016/j.jot.2020.12.005 33575166 PMC7844433

[B71] Valdes A. M. Spector T. D. (2011). Genetic Epidemiology of Hip and Knee Osteoarthritis. Nat. Rev. Rheumatol. 7 (1), 23–32. 10.1038/nrrheum.2010.191 21079645

[B72] van der Kraan P. M. (2017). The Changing Role of TGFβ in Healthy, Ageing and Osteoarthritic Joints. Nat. Rev. Rheumatol. 13 (3), 155–163. 10.1038/nrrheum.2016.219 28148919

[B73] Vasheghani F. Zhang Y. Li Y. H. Blati M. Fahmi H. Lussier B. (2015). PPARγ Deficiency Results in Severe, Accelerated Osteoarthritis Associated With Aberrant mTOR Signalling in the Articular Cartilage. Ann. Rheum. Dis. 74 (3), 569–578. 10.1136/annrheumdis-2014-205743 25573665 PMC4345902

[B74] Weber D. Wiese C. Gessler M. (2014). Hey BHLH Transcription Factors. Curr. Top. Dev. Biol. 110, 285–315. 10.1016/B978-0-12-405943-6.00008-7 25248480

[B75] Wu Z. Nagata K. Iijima T. (2002). Involvement of Sensory Nerves and Immune Cells in Osteophyte Formation in the Ankle Joint of Adjuvant Arthritic Rats. Histochem. Cel Biol. 118 (3), 213–220. 10.1007/s00418-002-0443-x 12271357

[B76] Xu J. Jiang C. Zhu W. Wang B. Yan J. Min Z. (2015). NOD2 Pathway via RIPK2 and TBK1 Is Involved in the Aberrant Catabolism Induced by T-2 Toxin in Chondrocytes. Osteoarthritis Cartilage. 23 (9), 1575–1585. 10.1016/j.joca.2015.04.016 25917637

[B77] Yang H. Wen Y. Zhang M. Liu Q. Zhang H. Zhang J. (2020). MTORC1 Coordinates the Autophagy and Apoptosis Signaling in Articular Chondrocytes in Osteoarthritic Temporomandibular Joint. Autophagy. 16 (2), 271–288. 10.1080/15548627.2019.1606647 31007149 PMC6984599

[B78] Yang S. Kim J. Ryu J. H. Oh H. Chun C. H. Kim B. J. (2010). Hypoxia-Inducible Factor-2Alpha Is a Catabolic Regulator of Osteoarthritic Cartilage Destruction. Nat. Med. 16 (6), 687–693. 10.1038/nm.2153 20495569

[B79] Yang S. Ryu J. H. Oh H. Jeon J. Kwak J. S. Kim J. H. (2015). NAMPT (Visfatin), a Direct Target of Hypoxia-Inducible Factor-2α, Is an Essential Catabolic Regulator of Osteoarthritis. Ann. Rheum. Dis. 74 (3), 595–602. 10.1136/annrheumdis-2013-204355 24347567 PMC4345811

[B80] Yano F. Hojo H. Ohba S. Fukai A. Hosaka Y. Ikeda T. (2013). A Novel Disease-Modifying Osteoarthritis Drug Candidate Targeting Runx1. Ann. Rheum. Dis. 72 (5), 748–753. 10.1136/annrheumdis-2012-201745 23041841

[B81] Yano F. Ohba S. Murahashi Y. Tanaka S. Saito T. Chung U. I. (2019). Runx1 Contributes to Articular Cartilage Maintenance by Enhancement of Cartilage Matrix Production and Suppression of Hypertrophic Differentiation. Sci. Rep. 9 (1), 7666. 10.1038/s41598-019-43948-3 31113964 PMC6529519

[B82] Yuan C. Pan Z. Zhao K. Li J. Sheng Z. Yao X. (2020). Classification of Four Distinct Osteoarthritis Subtypes With a Knee Joint Tissue Transcriptome Atlas. Bone Res. 8 (1), 38. 10.1038/s41413-020-00109-x 33298863 PMC7658991

[B83] Zhang F. J. Luo W. Lei G. H. (2015). Role of HIF-1α and HIF-2α in Osteoarthritis. Jt. Bone Spine. 82 (3), 144–147. 10.1016/j.jbspin.2014.10.003 25553838

[B84] Zhen G. Wen C. Jia X. Li Y. Crane J. L. Mears S. C. (2013). Inhibition of TGF-β Signaling in Mesenchymal Stem Cells of Subchondral Bone Attenuates Osteoarthritis. Nat. Med. 19 (6), 704–712. 10.1038/nm.3143 23685840 PMC3676689

[B85] Zhou Y. Zhou B. Pache L. Chang M. Khodabakhshi A. H. Tanaseichuk O. (2019). Metascape Provides a Biologist-Oriented Resource for the Analysis of Systems-Level Datasets. Nat. Commun. 10 (1), 1523. 10.1038/s41467-019-09234-6 30944313 PMC6447622

[B86] Zhu X. Chen F. Lu K. Wei A. Jiang Q. Cao W. (2019). PPARγ Preservation via Promoter Demethylation Alleviates Osteoarthritis in Mice. Ann. Rheum. Dis. 78 (10), 1420–1429. 10.1136/annrheumdis-2018-214940 31239244

